# Aging village doctors in five counties in rural China: situation and implications

**DOI:** 10.1186/1478-4491-12-36

**Published:** 2014-06-28

**Authors:** Huiwen Xu, Weijun Zhang, Linni Gu, Zhiyong Qu, Zhihong Sa, Xiulan Zhang, Donghua Tian

**Affiliations:** 1 China Institute of Health, School of Social Development and Public Policy, Beijing Normal University, 19 Xinjiekou Wai Street, Haidian District, Beijing, China

**Keywords:** Aging, Barefoot doctors, China, Rural health workforce, Village doctors

## Abstract

**Background:**

The aging population, rapid urbanization, and epidemiology transition in China call for the improvement and adaptation of the health workforce, especially in underserved rural areas. The aging of village doctors (the former “barefoot doctors”) who have served the rural residents for many decades has become a warning signal for the human resources for health in China. This study aims to investigate the village doctors’ aging situation and its implications in rural China.

**Methods:**

The data reviewed were obtained from the baseline survey of a longitudinal study of rural health workforce in five counties in rural China in 2011. Using a stratified multi-stage cluster sampling process, the baseline data was collected through the self-administered structured Village Doctor Questionnaire. Descriptive analyses, correlation analyses, and multivariate linear regression with interaction terms were conducted with the statistics software Stata 12.0.

**Results:**

The average age of the 1,927 village doctors was 49.3 years (95% CI 48.8 to 49.9), 870 (45.2%) of whom were aging (50 years or older). Both the age and the recruitment time of the village doctors were demonstrated to have a bimodal distribution. A greater proportion of the male village doctors were aging. Furthermore, aging of the village doctors was significantly correlated to their education level, type of qualification, practicing methods, and their status as village clinic directors (*P* <0.05, respectively). As shown in the regression models, aging village doctors provided significantly more outpatient services to rural residents (*P* <0.01) but without an increase in income, and their expected pension was lower (*P* <0.01), compared with their non-aging counterparts.

**Conclusions:**

Aging of village doctors is a serious and imperative issue in China, which has a complex and profound impact on the rural health system. Greater attention should be paid to the construction of the pension system and the replenishment of the village doctors with qualified medical graduates.

## Background

The population of China is aging rapidly due to the cumulative effects of the consistently low fatality rate [1], the one-child policy [2], and the increasing life expectancy [1]. The proportion of the elderly population (aged over 65 years) of China is projected to reach 23% to 30% by 2050, as estimated by the United Nations [1,3]. At the same time, China is urbanizing at a rapid rate due to the rural-to-urban migration occurring since the post-1978 economic reform. The urban population rose from 191 million in 1980 to 622 million in 2009, and the urban population further overtook the rural population in 2011 (51% vs. 49%) [4]. As more than 85% of the migrates are younger than 40 years old [5], migration has greatly accelerated the population aging in the rural areas – by 2050, the population aging in rural areas is estimated to be more severe than that in urban areas when taking the rural-to-urban migrates into consideration (23% vs. 21%) [6].

The interaction between population aging and fast urbanization exerts increased pressure on the health system in rural China. Firstly, due to the health migrate phenomenon, people migrating to urban districts are relatively healthier than those who do not [7]. Therefore, the remaining rural residents, mainly the elderly and the very young, have greater healthcare demands than the migrating population. Secondly, when migrating workers fall ill or become injured, they always return to their hometown to receive healthcare services, probably due to the fragment of the health insurance system and the high out-of-pocket expenditure in urban hospitals [4,8]. Thus, the healthcare demands of rural China have not decreased in parallel with the decrease in the rural population; on the contrary, such a transition requires rural health facilities to provide more and better healthcare to the remaining rural residents.

Along with population aging, the last 30 years have also seen an epidemiology transition partly because of the rapid lifestyle changes occurring within this period. In 2005, chronic diseases accounted for 80% of deaths and 70% of the disability-adjusted life-years in China [9]. In rural China, the three main causes of death (i.e., malignancy, cardiac diseases, and cerebrovascular diseases) accounted for over 60% of all deaths in 2011. Thus, chronic diseases have overcome communicable diseases to become the major disease burden in China [10]. Specifically, the prevalence of hypertension in rural adults was 31.3%, slightly higher than that in urban adults (29.2%) [11]. For diabetes, the prevalence in rural areas was lower than that in urban areas (10.3% vs. 14.3). However, the prevalence of pre-diabetes in rural residents was higher than that in urban residents (50.9% vs. 48.4%), whereas less diabetes cases were diagnosed in rural areas compared to urban areas (2.5% vs. 5.6%) [12]. Thus, if no effective intervention is implemented in rural districts, the prevalence of diabetes in rural areas might overtake that of urban areas in the near future [13].

To meet these challenges, the rural health system needs to be more effective and efficient, and the human resources for health should be improved not only in quantity, but also with regards to equitable distribution, competency, quality, motivation, productivity, and performance [14]. The health system in rural China has a three-tiered structure: county hospitals, township health centers (THCs), and village clinics [15]. The county hospitals and THCs are usually of public ownership and managed by local health bureaus. For physicians working at the THCs and hospitals, they must pass the national Licensed Doctors Examination or the Licensed Assistant Doctors Examination after at least three years’ medical education at a medical college. As they are publicly employed, they have an institute-based income and public pension provided by the government. Furthermore, doctors from the public hospitals and THCs must retire and receive a pension at the age of 60 for males and 55 for females.

The establishment of village clinics differs from that of THCs and public hospitals. Before 1980, the village clinics were supported by the village collective economy. After the collapse of the collective economy following the economic reforms, some village clinics were supported by the village committees or township governments, while others were supported by the village doctors themselves, who became private practitioners and made money mainly through the selling of drugs [16,17]. Recently, the Chinese government has begun to once again fund the construction of village clinics, aiming to establish a standard clinic in each village. Moreover, the village doctors were previously not included in the public pension system, since they were registered as farmers rather than public employees. Therefore, the pension they would receive from the New Rural Social Pension Insurance System, a new universal pension scheme for all rural elderly in effect since 2009, amounted to 55 RMB per month [18]. Recently, some counties, such as Changshu and Yongchuan, have launched a special pension for their retired village doctors [16].

Medical education in China has also changed considerably during the past decades [19]. Before 1980, the main purpose of the medical education system was to provide sufficient health professionals to the rural villages in order to tackle the wide urban-rural disparity. After 1980, China prioritized the steady development of its medical universities to provide sufficient and qualified doctors to the health system overall [19]. In 1998, China expanded its tertiary education system after a major expansion of comprehensive universities, which led to the rapid growth of health professionals [20]. However, the human resources for health were not distributed according to the needs in rural and urban areas – doctor density in the urban areas was more than twice that in the rural areas [20]. In the villages, about one million village doctors still played an important role in providing healthcare to rural residents. The minimum requirement for medical practice at the village clinics was to have passed the local examination held by the county health bureau and to obtain the Village Doctor Certification, while some village doctors could also pass the National Licensed (Assistant) Doctors Examination and become a licensed (assistant) doctor. By 2010, only a minority (14.2%) of the village doctors had passed the national examination [21].

A proportion of the village doctors remain from the barefoot doctors, who have been practicing medicine for over 40 years in rural China; these doctors will inevitably retire within the next few decades [16,17]. Earlier studies on barefoot doctors did not take the aging factor into consideration [22-28]. The age structure of the village doctors has changed considerably over time, and has begun to skew towards the aging side in recent years. Several recent studies have shown that more than 30% of the village doctors in the sample areas (both developed and under-developed districts) were 50 years old or older in 2009, and few village doctors were younger than 30 years old [29-34]. However, these studies had some limitations. First, their sampling methods were various, including multistage sampling [29,31], convenience sampling [35], multistage random cluster sampling [30], and cluster sampling [33]. Furthermore, several studies did not report the sample population and the response rate [29,30,32], and the quality control was unclear [29-33], leading to inconvincible results. Additionally, all these studies were descriptive, only reporting the average age or the percentage of each age group, while the causes and implications of the aging of village doctors were not deeply discussed.

Recently, there have been several new policy progresses regarding the village doctors, mainly including improving the quality, strengthening the management, and prioritizing public health services [36]. First, to improve the quality of the provision of village doctors, their on-the-job training has become a requirement by the central government. Second, the government began to monitor the village clinics with a more rigorous standard, especially for the certification of village doctors, the use of drugs, and the reimbursement procedure. Third, since the health reform in 2009, the Ministry of Health (MOH) has requested the village doctors to provide both medical and public health services to the rural residents [21]. As a matter of fact, there was still no consistent or sufficient public funding for the operation of the village clinics, although the village doctors could receive some compensation through various channels [16].

Therefore, more systematic analyses regarding the aging of the village doctors are required in order to benefit the rural health system in China, since village doctors provided 1.59 billion outpatient services for rural residents in 2011 [13]. If the aging challenge is not addressed, it will probably result in a human resource for health crisis in rural China in the near future. This study aimed to reveal the age structure of the village doctors and to explore the causes and the relationship between aging and education levels, practicing methods, outpatient numbers, medical income, and expected pension. The outpatient numbers per month, implying the average quantity of outpatients treated by a single village doctor, were included into the analysis to gauge the workload and the capacity of the village doctors. As the income and the retirement pension were considered to be the most important factors of the recruitment and retention of village doctors in Beijing [34], we further conducted quantitative analysis on them. Therefore, this study tried to present a panorama of the aging of the village doctors in China.

## Methods

### Study population and design

The data was obtained from the baseline survey of a longitudinal study of rural health workforce in five counties in China since 2011, conducted by the China Institute of Health and approved by the School of Social Development and Public Policy Ethics Committee at the Beijing Normal University [16]. The self-administered structured Village Doctor Questionnaire, modified from the official questionnaire from the MOH after three focus groups, in-depth interviews in Hebei Province, and a pilot survey in Sichuan Province, was the tool to investigate the village doctors in this study [16].

The stratified multi-stage cluster sampling method was used to select the sample. During the first stage, Jiangsu Province (southeast), Sichuan Province (southwest), Chongqing Municipality (middle-west), and Gansu Province (northwest) were selected from the 34 provinces or municipalities of China. Second, one or two counties or districts were chosen from each province or municipality: Changshu County and Liyang County from Jiangsu, Mianzhu County from Sichuan, Yongchuan District from Chongqing, and Jingning County from Gansu [16]. Although the geographic distribution and socioeconomic status were taken into account in the sampling process, the initial two steps of sampling were not random. The third stage involved obtaining a list of all village doctors in each county from the local health bureaus. All of the identified village doctors were asked to participate in this survey, and those who agreed to participate in the survey were given a written informed consent. The questionnaires were independently finished by the village doctors after the illustration of the objectives of the survey by 10 trained interviewers in each sampling county. Overall, 1,982 of 2,250 village doctors participated in the survey, and the response rate was 88.1%. More detailed information about the research design was discussed elsewhere [16].

### Variables

Age and a dichotomous variable “aging” which referred to the village doctors aged 50 years or older were included into the analysis, while the non-aging doctors were those younger than 50 years old. Fifty years was used as the cut-off value based on previous studies [29-33], which was the standard medical classification of old age. Furthermore, China mandates retirement at 60 years for males and 55 for females. “The year of becoming a village doctor” was used to illustrate the dynamic workforce supply of the village doctors, which might be helpful for explaining the cause of aging.

To compare the basic characteristics between aging and non-aging village doctors, we utilized the following variables: the highest education obtained (i.e., junior high school or less, high school, secondary school, junior college, and college or above), the practicing methods (western medicine, traditional Chinese medicine, or mixed methods), the qualification (licensed (assistant) doctors, or village doctors), method of obtaining the highest degree (regular training or on-the-job training), and status as director of the village clinic (yes or no). All of those variables were consistent with the official statistics, and had been used in the previous study [16].

Furthermore, to explore the implications of aging, outpatient numbers per month, basic medical income per month, and self-reported expected pension per month were used as dependent variables in the final multivariate linear regression models. Specifically, the outpatient numbers and the basic medical income was reported by village doctors themselves. The basic medical income was mainly the profits from medical services, not including the public health subsidy from the government, or income from the agricultural activities of those village doctors with farmlands [16]. The expected pension was measured by the question “What is your expected pension per month after you retire?” which was the self-reported expectation about pension, rather than what they could receive in the current pension scheme for rural residents (i.e., 55 RMB per month). As these dependent variables might also be influenced by their gender, qualifications, etc., those basic characteristics were also included in the multivariate models, except for the practicing method, which was not significant in the univariate and multivariate regression models. In addition, considering the huge disparities in socio-economic development of the sampling counties [16], a categorical variable “county” was incorporated into the models.

### Statistical analysis

Descriptive analyses were conducted to show the age structure of the village doctors in sample counties, and the correlation analyses were performed to show the relationship between basic characteristics and aging. Then linear regressions were conducted to show the associations between aging and outpatient numbers, basic medical income, and expected pension. First, three univariate linear regression models were built, after which three multivariate linear regressions were constructed. As shown in Table 1, the dependent variables were outpatient numbers, basic medical income, and expected pension, respectively. The independent variables included gender, aging, highest education obtained, qualification, method of earning the highest degree, and status as director of the village clinic. The above independent variables were recoded into dichotomous variables as described in Table 1.

**Table 1:** Description of variables used for multivariate regression analyses

	**N**	**Mix/Max**
**Dependent variables**		
Outpatient numbers (per month)^a^	1,474	10–1,205
Basic medical income (per month)^b^	1,664	15–40,000
Expected pension (per month)^c^	1,530	100–10,000
**Independent variables**		
Male (vs. female)	1,927	0–1
Ageing (vs. non-ageing)	1,927	0–1
Junior college or above (vs. secondary school or below)^d^	1,927	0–1
Licensed (assistant) doctors (vs. barefoot doctors)	1,927	0–1
Director of village clinics (vs. non-director)	1,927	0–1
Prior to employment (vs. on-the-job training)	1,800	0–1
County	1,927	–
Changshu	333	0–0
Liyang	316	0–1
Yongchuan	588	0–1
Mianzhu	358	0–1
Jingning	332	0–1

^a^Village doctors’ self-reported monthly workload.

^b^Profits from the medical services, not including the public health subsidy and income from others channels (agriculture and so on).

^c^Self-reported excepted pension which can fulfill their basic living standard.

^d^To simplify the analysis, junior high school or less, high school, and technical secondary school were classified as technical secondary school or below; junior college and college or above were classified as junior college or above.

As the sample in our study was chosen from counties with different economic development levels, it would be better to include the county level index and to perform the multilevel analysis. However, only five counties (groups) were selected, which was not suitable for multilevel analysis [37]. Instead, we introduced a categorical variable “county” into the analysis, and constructed an interaction term: aging*county, to show the interaction effects of aging and geography in the multivariate models. All the statistics analyses were performed by using Stata Version 12.0 (StataCorp LP, College Station, Texas, USA).

### Missing data

As the main purpose of this study was to explore the implication of the aging on the village doctors, questionnaires without the information of age were excluded. Finally, 1,927 questionnaires were included in this study. The missing data were shown as follows: outpatient number (23.5% missing), basic medical income (13.7% missing), and expected pension (20.6% missing), the method of obtaining the degree (6.6% missing), and the number of years working as a village doctor (4.1% missing). There were no missing data among all other variables in our analysis. The list-wise approach was employed to deal with the missing data in all the statistics analyses.

## Results

### The mean age and aging of village doctors

As shown in Table 2, the mean age of the 1,927 village doctors was 49.3 years; and 870 (45.2%) of them were 50 years old or older. The age structure was not constant in different counties, as the mean ages were 50.6 years in Changshu, 55.1 years in Liyang, 51.2 years in Mianzhu, 47.3 years in Yongchuan, and 44.2 years in Jingning. The proportion of aging village doctors also varied in the different counties, and were 57.4% in Changshu, 67.7% in Liyang, 52.8% in Mianzhu, 35.4% in Yongchuan, and 20.5% in Jingning.

**Table 2:** The mean and median age, and aging of village doctors by sample counties in years

**County**	**Mean**	**Median**	**95% CI**	**N (%)**
Changshu	50.6	54	49.3–51.8	191 (57.4)
Liyang	55.1	58	52.9–56.3	214 (67.7)
Mianzhu	51.2	53	49.8–52.6	189 (52.8)
Yongchuan	47.3	44	46.3–48.3	208 (35.4)
Jingning	44.2	43	43.1–45.3	68 (20.5)
Total	49.3	47	48.8–49.9	870 (45.2)

### The age structure and starting work year of village doctors by gender

**Figure 1:**
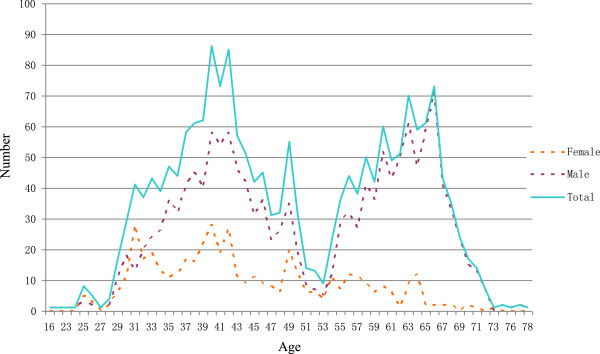
Age distribution of village doctors by gender.

**Figure 2:**
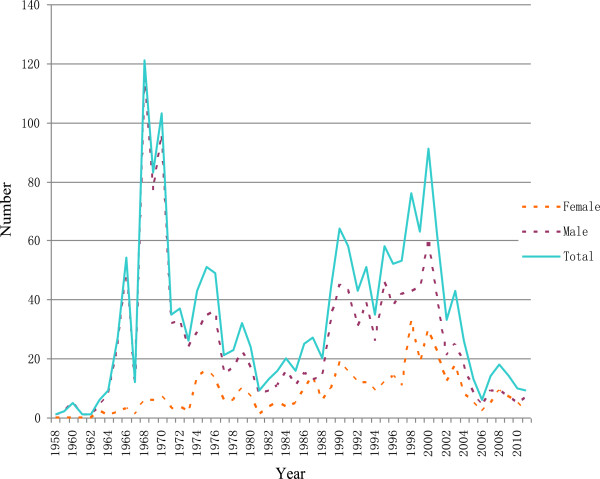
The year of becoming village doctors by gender.

There was significant difference in age structure between female and male village doctors, as shown in Table 3 and Figure 1. Overall, female village doctors were younger than males. More than half of the female village doctors were aged 30–49 years (68.0%), while most of male village doctors were aged 30–49 years (48.0%) or older than 60 years old (32.1%). For both males and females, few of them were younger than 30 years old. The bimodal distribution of the age structure of the village doctors is shown in Figure 1.Similarly, Figure 2 shows the distribution of the year in which participants began to work as village doctors, which was an indication of workforce supply in rural areas. There was a rapid increase of the village doctors in the 1960s, followed by a sharp decrease in the late 1970s, a resurge in the 1980s and 1990s, and then a sharp fall again in the 2000s. This indicate that most village doctors entered the rural health workforce between the years 1966 and 1976 or between the years 1986 and 2004, and only 84 (4.6%) began to work as village doctors after the year 2006. It is obvious that the fluctuation in age distribution of the village doctors in Figure 1 was associated with the fluctuation of workforce supply in Figure 2.

**Table 3:** Age distributions among female and male village doctors

**Age**	**Females (%)**	**Males (%)**	**Total (%)**
<30	16 (3.5)	23 (1.6)	39 (2.0)
30–49	314 (68.0)	704 (48.0)	1018 (52.8)
50–60	92 (19.9)	268 (18.3)	360 (18.7)
>60	40 (8.6)	470 (32.1)	510 (26.5)
Total	462 (100)	1465 (100)	1927 (100)

### Aging and basic characteristics of the village doctors

Table 4 demonstrates the basic characteristics of the village doctors by age group. Compared with the aging village doctors, the non-aging village doctors were more likely to have higher education levels (*P* <0.01), national registration (*P* <0.01), be directors of village clinics (*P* <0.01), and less likely to practice both western and traditional medicine (*P* = 0.036). The stratification analysis in Table 5 shows the method of obtaining their highest degree played a role in the difference in the type of qualification between aging and non-aging village doctors. Non-aging village doctors who obtained degrees before being hired were more likely to pass the licensed doctor examination than their aging counterparts (*P* = 0.001), while no difference existed between aging and non-aging village doctors who obtained their degrees after being hired (*P* = 0.22).

**Table 4:** Comparison of characteristics between aging and non-aging village doctors

	**Non-aging**^**a **^**(%)**	**Aging**^**b **^**(%)**	**All (%)**	***P *** **value**
**Education**				<0.001
Junior high school or less	29 (2.7)	402 (46.2)	431 (22.4)	
High school	48 (4.5)	127 (14.6)	175 (9.1)	
Secondary school	837 (79.2)	315 (36.2)	1152 (59.7)	
Junior college	138 (13.1)	26 (3.0)	164 (8.5)	
College or above	5 (0.5)	0 (0.0)	5 (0.3)	
**Qualification**				<0.001
Licensed (assistant) doctors	260 (24.6)	154 (17.7)	414 (21.5)	
Village doctors	797 (75.4)	716 (82.3)	1,513 (78.5)	
**Practicing methods**				0.036
Western medicine	437 (41.3)	311 (35.8)	748 (38.8)	
Traditional Chinese Medicine	31 (2.9)	24 (2.8)	55 (2.9)	
Mixed methods	589 (55.7)	535 (61.4)	1,124 (58.3)	
**Director of village clinics**				<0.001
Non-directors	471 (44.6)	497 (57.1)	968 (50.2)	
Directors	586 (55.4)	373 (42.9)	959 (49.8)	
**All**	1,057 (100)	870 (100)	1,927 (100)	

^a^Non-ageing refers to the village doctors younger than 50 years old.

^b^Ageing refers to village doctors who are 50 years old or above.

**Table 5:** Cross-table of age group and professional qualifications by training methods

	**On-the-job training**^**a **^**N (%)**		**Prior to employment**^**b **^**N (%)**	
**Age**	**Licensed (assistant) doctors**^**c**^	**Village doctors**^**d**^	**Licensed (assistant) doctors**	**Village doctors**
<50	116 (59.2)	456 (54.9)	139 (70.5)	318 (55.2)
50–60	28 (14.3)	163 (19.6)	21 (10.7)	105 (18.2)
>60	52 (26.5)	212 (25.5)	37 (18.8)	153 (26.6)
Total	196 (100)	831 (100)	197 (100)	576 (100)
*χ*^2^	3.01		14.63	
*P* value	0.221		0.001	

^a^On-the-job training refers to village doctors obtaining their highest education degree through the on-the-job training system held by the county government.

^b^Prior to employment refers to village doctors obtaining their highest education degree before working, which means that they received regular training in the medical school.

^c^Doctors who passed the national medical licensing examination get the certification to practice.

^d^Unlicensed (assistant) doctors usually received the Village Doctor Certification from the county government.

### The implications of aging on the village doctors

The variables used in the regression models are described in Table 1. Table 6 shows the final results of the regression analyses. In the univariate models, it is found that aging was reversely associated with outpatient numbers (*P* <0.05) and the expected pension (*P* <0.01), but not significantly correlated with the basic medical income.

**Table 6:** Multivariate regression models of village doctors by outpatient numbers, basic medical income (RMB per month), and expected pension (RMB per month)

	**Outpatient numbers, SE**	**Basic medical income, SE**	**Expected pension, SE**
**Univariate model**			
Aging (Ref: non-aging)	−20.8 (9.3)**	80.0 (87.0)	−363.7 (51.5)***
Constant	211.7 (6.2)	1778.7 (58.3)	2147.2 (34.8)
**Full model**			
Male (Ref: female)	24.5 (11.0)**	274.9 (92.5)***	21.9 (58.8)
Aging (Ref: non-aging)	54.6 (20.8)***	−100.4 (171.2)	−415.3 (115.2)***
Junior college or above (Ref: secondary school or below)	28.5 (14.9)	233.1 (128.9)	−25.4 (85.1)
Licensed (assistant) doctors (Ref: village doctors)	18.6 (10.8)	−26.8 (90.0)	51.2 (57.0)
Director of clinics (Ref: non-director)	28.0 (9.4)***	100.7 (79.6)	−12.1 (50.9)
Prior to employment (Ref: on-the-job training)	−18.7 (8.5)**	−104.1 (73.2)	−63.6 (47.4)
County (Reference: Changshu)			
Liyang	−127.5 (25.1)***	454.8 (188.3)**	−523.2 (122.9)***
Yongchuan	−113.2 (18.8)***	−343.0 (153.4)**	−14.6 (101.6)
Mianzhu	62.1 (20.6)***	−1129.3 (169.9)***	−932.4 (113.7)***
Jingning	60.8 (20.4)***	−1405.2 (180.3)***	−1459.8 (119.6)***
Aging* County (Reference: aging* Changshu)			
Aging* Liyang	−56.1 (31.1)	−413.4 (239.1)	−153.6 (156.9)
Aging* Yongchuan	−51.4 (26.0)**	−208.5 (214.8)	−234.3 (142.5)
Aging* Mianzhu	−140.0 (27.6)***	−338.5 (239.2)	141.8 (155.4)
Aging* Jingning	−99.2 (32.5)***	10.8 (318.4)	494.6 (201.6)***
Constant	199.7 (16.4)	2124.3 (131.9)	2659.8 (89.1)
Adjusted R^2^	0.24	0.14	0.26

**P <0.05; ***P <0.01.

In the multivariate regression model for outpatient numbers (Model 1), the aging village doctors were more likely to provide more outpatient services than the non-aging village doctors (*β* = 54.6, *P* <0.01). Interaction analysis revealed that compared with non-aging doctors from Changshu, those from Liyang and Yongchuan had lower outpatient numbers, while those from Mianzhu and Jingning provided more outpatient services. Aging village doctors from Yongchuan, Mianzhu, and Jingning had less outpatient numbers than those from Changshu, while no significant difference was found between Liyang and Changshu. Being male or directors of village clinics were positively associated with outpatient visits per month; whereas, receiving regular training was negatively correlated with the outpatient numbers.

In Model 2 for basic medical income, no significant differences in basic medical income were confirmed between the aging and non-aging village doctors, which was opposite to the result in the univariate model. In addition, the non-aging village doctors from Liyang had a higher basic medical income than from Changshu. In contrast, the village doctors from Yongchuan, Mianzhu, and Jingning earned significantly less than those from Changshu. No significant difference in basic medical income was observed among the aging doctors in different counties. For other covariates, only being male was significant in the regression model (*P* <0.01).

Model 3 presents the multivariate regression with the expected pension as the dependent variable. The expected pension from the aging village doctors was 415.3 RMB lower than that from non-aging doctors (*P* <0.01). Furthermore, non-aging village doctors from Liyang, Mianzhu, and Jingning expected lower pensions than those from Changshu (all *P* <0.01). The aging village doctors from Jingning had a higher expected pension (i.e. 494.6 RMB per month) than those from Changshu (*P* <0.01), while no significance was found among other groups. No significant difference was found among other independent variables in Model 3.

## Discussion

### The fluctuating workforce supply of the village doctors in China

In this study, it was found that the aging of village doctors in China is serious and imperative, especially in the developed counties of Changshu and Liyang, which was consistent with the findings from other studies [30,31,35]. The aging of the village doctors was partly induced by the large fluctuation of the workforce supply for rural health during the past decades. Coming from the barefoot doctors, which was a political creation in 1970s [38], they have been strongly influenced by the government policies since then. In our study, most participants began to practice medicine between 1966 and 1976 or between 1988 and 2004. The first surge (1966 to 1976) was due to the initiation of the barefoot doctors, when approximately 4 million people were trained [39]; the second peak occurred between 1988 and 2004, as the MOH trained many people to pass the examination in order to tackle the health workforce crisis following the drop of village doctor numbers to 643,022 in 1985 [17,39].

Few village doctors were trained after 2006. At that moment, China began to prioritize the education of qualified physicians with international standards [19], thus less efforts were made to train village doctors. In addition, China’s health reform starting from the year 2009 may have influenced the working motivation of the village doctors, as they were required to provide time-consuming public health services to rural residents with few subsidies [21] and to sell the drugs without profit [16]. Those factors conjointly reduced the income of the village doctors, and some village doctors even changed their occupation following the reform [40]. Although three funding streams (both central and local) were built to compensate the income loss [21], the local government lacked the proper incentives or sufficient financial capacity to provide such funding [40,41]. As a result, young medical graduates were unwilling to enter the rural health system.

### The differences between aging and non-aging village doctors

Aging village doctors were less educated, preferred mixed practicing methods, and were less likely to be directors of village clinics than younger doctors. The development of public education in China [42] provided better opportunities for the young village doctors, and thus they had a much higher educational level than aging doctors. Additionally, the medical education of village doctors has changed throughout the years. At first, barefoot doctors only received several months of training in a mixture of Traditional Chinese Medicine and western medicine [17], resulting in aging village doctors preferring mixed methods, while young village doctors had more opportunities to receive regular training at medical schools [19], and thus preferred western medicine. Although aging doctors had worked longer than non-aging village doctors, it did not ensure them being appointed as directors of village clinics, since the appointment is determined by many factors such as their relationship with the THCs and the capacity to accomplish the public health and other workloads allocated by local governments.

### The impact of aging on the services, income, and expected pension

The aging village doctors provided significantly more outpatient services to rural residents than their non-aging counterparts, partly because of their abundant clinical experience and mutual trust between them and their patients. This identified the service abilities of the aging village doctors. In other words, the aging village doctors still played an important role in the rural health system. From this perspective, the retirement of the aging workforce could be a real challenge for the health system if no sufficient young workforce is supplemented into the system.

With regards to the basic medical income, no significant difference was found in the multivariate regression model, which means aging village doctors did not earn more than non-aging doctors. Considering the higher number of outpatient services provided by aging village doctors, it could be inferred that the average charge by aging village doctors should be lower than that by non-aging doctors. This is probably due to aging village doctors providing less complicated services, or selling fewer drugs (their income is decided by the amount of drugs they sell), or other such reasons. Furthermore, the non-aging village doctors from Changshu and Liyang earned more than those from Yongchuan, Mianzhu, and Jingning, regardless of the higher outpatient numbers from Mianzhu and Jingning. In contrast, the incomes of the aging village doctors from different counties showed no difference. It might be concluded that the economic development was a decisive factor for non-aging village doctors’ income, as reported in previous studies [29-31]; however, aging village doctors were less influenced by the local economics. If the pension system is designed to compensate for the income loss of the aging village doctors, the effect of the local economic development level would not be as important as previously thought [16].

It was found that non-aging village doctors expected a higher level of pension, which indicated that they might consider a greater inflation and overall rise of living standards in the future. In addition, non-aging village doctors from counties with worse economic situations had a much lower expected pension, as much as three-fold of the per capita net income of rural residents in 2011 [43], implying that the expected pension was highly influenced by local living costs and consumption levels. However, the aging village doctors from different areas had a relatively consistent expected pension, with those from Jingning as an exception. This implicated that a one-site-fit-all pension standard for the aging village doctors in China is perhaps possible.

### The implications of the aging village doctors for China’s rural health system

The aging of the village doctors will have multiple impacts on China’s rural health system. Firstly, the large fluctuation in the rural health workforce supply indicates a risk of workforce shortage at some stage in the near future. In fact, almost half of the doctors will retire in approximately 10 years’ time. If the supply of rural village doctors remains at the current low level, it is logical to assume that there will be a shortage of village doctors in 10 years’ time, which will perhaps impose a great pressure on China’s three-tiered rural health system.

Secondly, the characteristics associated with the aging village doctors will probably damage the accessibility of medical services of rural residents. The low education, the lack of formal training, and the difficulty of adapting to new technologies of the aging village doctors will deter rural residents from seeking qualified services from them. The epidemiological transition in rural China requires that the village doctors provide more long-term care for patients with chronic diseases and especially for the elderly, which is encouraged by the government [36]. Meanwhile, millions of children and females left behind in rural areas require maternal and child care services from the village doctors. However, aging village doctors lack training in these specialties and therefore experience great pressure to provide these services [17,44-46].

As the quality of services is significantly associated with the quality of the health workforce [47], the aging village doctors might reduce the quality of healthcare provided to rural residents, especially in services for non-communicable diseases. Conversely, the urban population can access high-quality services from the sufficiently well-trained doctors in the urban health system. As a consequence, the aging of the village doctors and the probable shortage of workforce will deteriorate the urban-rural equity on the quality of services, which has indeed been a continuing challenge in China since the 1980s [48,49]. To fix the gap, the aging village doctors would better be replaced by an adequately skilled, well-trained, and motivated workforce to guarantee the achievement of health equity and Universal Health Coverage in China [14,50].

Furthermore, the possible shortage in the health workforce related to the aging of the village doctors will also influence the performance of the health system in China. In order to obtain satisfactory medical services, rural residents must access the higher level institutions, mainly the THCs and county hospitals, resulting in the upward movement of the patients [51]. These alternative health institutions would take over the responsibility of the village doctors with regards to the provision of more services, and mainly outpatient services. Due to the higher cost of services at higher level health institutions [13] and other indirect costs such as transportation and the value of lost productivity from time off work, the gross health expenditure of China will probably be increased. In addition, due to the relatively high out-of-pocket health expenditure of the rural residents [52], the economic burden for rural residents when seeking medical services will eventually be raised to unsurmountable levels.

Finally, the shortage in village doctors might further impede the progress of China’s ambitious health reform, as the village doctors are currently responsible for conducting large quantities of public health services at the village level such as maintenance of health records, managing chronic diseases, health education, etc. [36]. Moreover, the raised expenditure of medical services due to the shortage of village doctors might increase the incidence of catastrophic health expenditure and household impoverishment in rural areas, which are also opposite to the goals of the healthcare reform [53,54].

### Policy suggestions to tackle the aging of the village doctors

Fortunately, the Chinese government has acknowledged the aging of village doctors and has launched policies to tackle the potential workforce shortage. Actions, such as recruiting graduates from medical universities to be the village doctors in Hebei Province [55] or training high school graduates to be the village doctors in Zhejiang Province [56], have shown the positive signs of these policies. Actually, the recruitment is not a big problem, as only 30% of medical university graduates enter the healthcare system in China [19]. Their retention, however, challenges the rural health managers: the young village doctors always leave their positions after the contract expiration, and it is difficult to find a substitute. Additionally, in the past, the majority of village doctors came from the local population with a good reputation, and mostly provided 24-h medical care services for the rural residents. Thus, these young medical graduates may change the village doctor culture since they do not provide night or out-of-clinic services.

In order to retain the young village doctors in China, an attractive income and retirement pension might be the critical factors [34,57,58], mainly because of the currently relatively low income and lack of retirement pension for the village doctors [16,34]. The government should also construct a safe and comfortable working environment, access to better training, career development opportunities, and a better education for their children [58-61]. Furthermore, considering the special cultural and social factors in rural China, the government would better recruit indigenous medical students with a rural background, so they will have a better social network to adapt the local life and enjoy the respect from the community [57,60]. With the economic and non-economic incentives, the young graduates will probably practice at the village clinics for a long period.

### Aging rural health workforce as an international issue

The aging trend of the village doctors resembles the situation of general practitioners or primary care physicians in developed countries such as Canada [62], the United States [63], and Australia [64], while there is a fundamental difference between the village doctors in China and primary care physicians in developed countries. The majority of the village doctors in China are underqualified [21], having only received limited training [17], whereas there are strict qualification requirements for primary care physicians in developed counties. It is true that the developed countries are facing serious workforce shortages, while China is facing an over-supply of medical graduates recently [19,20]. Therefore, China tries to supplement the loss of the aging rural health workforce through the recruitment of young graduates, while developed counties try to allow aging primary care physicians to choose part-time jobs or reduce their workloads [62,65,66], or recruit primary care physicians from developing countries [67,68].

### Strengths and limitations

The strengths of this study include its large number of subjects, broad geographic distribution, and deep discussions about the aging of the village doctors. However, the limitations should also be acknowledged. The selection of the counties and provinces was not random, and there were missing values in the survey. The indicators related to the medical service quality of the village doctors were not included in this study. Almost all variables in this study were related to the supply side (the village doctors), data from the demand side (the rural residents) was missing, and therefore the factors influencing outpatient numbers need to be further explored.

## Conclusions

China is facing serious challenges related to the aging of the village doctors, especially in developed areas. Therefore, more attention should be paid in addressing the workforce shortage due to the retirement of the aging village doctors. An appropriate nationwide pension system for the village doctors should be constructed as soon as possible in order to facilitate the replenishment process of the rural health workforce. To recruit and retain the qualified medical graduates into the rural health system, both economic and non-economic incentives should be implemented. In addition, similar to the barefoot doctors conditions in the 1970s, political support should be given to the rural health professionals.

In conclusion, the reconstruction of the rural health workforce would reinforce the basis of China’s health system, thus tackling the challenges of the aging population and the future rising numbers of patients with chronic diseases. The Chinese experience will be helpful for other developing countries to recognize the potential aging and shortage of the rural health workforce, and to make effective preventive strategies to cope with similar issues.

## Abbreviations

MOH: Ministry of health; THCs: Township health centers.

## Competing interests

We declare that we have no conflicts of interest.

## Authors’ contributions

DT, ZQ, XZ, ZS, and HX participated in the research design and project implementation. HX and WZ participated in the data collection and data analysis. HX wrote the original text. LG participated in the manuscript discussion and revision. All of the authors read and approved the final manuscript.
